# Posttraumatic growth of medical staff during COVID-19 pandemic: A scoping review

**DOI:** 10.1186/s12889-023-17591-7

**Published:** 2024-02-14

**Authors:** Qian Li, Yirong Zhu, Xuefeng Qi, Haifei Lu, Nafei Han, Yan Xiang, Jingjing Guo, Lizhu Wang

**Affiliations:** Department of Nursing, The Second Affiliated Hospital of Zhejiang University School of Medicine, No. 88 Jiefang Road, Shangcheng District, Hangzhou, 310009 Zhejiang Province China

**Keywords:** COVID-19, Posttraumatic growth, Psychological, Medical staff

## Abstract

**Background:**

The COVID-19 pandemic has imposed unprecedented stress and challenges upon medical staff, potentially resulting in posttraumatic growth (PTG). This scoping review aims to synthesize the existing knowledge on PTG among medical staff during the pandemic by identifying its current status and potential influencing factors. The findings may provide a foundation for future research and interventions to enhance the medical staff’s psychological resilience and well-being.

**Methods:**

Literature was systematically searched on PTG among medical staff during the COVID-19 pandemic from 01 January 2020 to 31 December 2022. The following databases were searched: PubMed, Web of Science, Embase, CINAHL, PsycINFO, Cochrane Library, China National Knowledge Infrastructure (CNKI), Chinese Biomedical Literature Service System (SinoMed), and Wanfang Data. Eligibility criteria included: (1) medical staff as research subjects; (2) a focus on “posttraumatic growth” or “alternative posttraumatic growth” related to the COVID-19 outbreak and pandemic; (3) discussion of the situation and influencing factors of PTG; and (4) study types, such as qualitative, quantitative, and mixed methods. Two researchers independently selected and extracted study characteristics (study design, study population, region, measurement instruments, and primary outcomes) from the included literature. The data were synthesized qualitatively and descriptively.

**Results:**

Thirty-six papers from 12 countries met the inclusion criteria. Moderate PTG levels were observed among healthcare workers during the COVID-19 pandemic, with emphasis on “interpersonal relationships,” “changes in life philosophy,” and “growth in personal competence.” Influencing factors included trauma exposure, sociodemographics, psychological characteristics (resilience and positive qualities), coping, and social support.

**Conclusions:**

This review discovered moderate PTG levels among medical staff during the COVID-19 pandemic, with critical areas in interpersonal relationships, life philosophy, and personal competence. The identified influencing factors can inform future research and interventions to enhance healthcare workers’ psychological resilience and well-being.

**Supplementary Information:**

The online version contains supplementary material available at 10.1186/s12889-023-17591-7.

## Introduction

Posttraumatic growth (PTG) has been defined as “positive psychological change that occurs following a struggle with highly challenging life circumstances” and through establishing perspectives for a “new normal” when the old normal is no longer an option [1]. The positive transformation developed five domains: development of deeper relationships, openness to new possibilities, a greater sense of personal strength, a stronger sense of spirituality, and a greater appreciation of life; followed by the development of the Posttraumatic Growth Inventory (PTGI), which has been translated into more than 20 languages and extensively validated worldwide [1, 2]. PTG is associated with PTGI across numerous cultures and many different traumatized populations, including those who have survived natural disasters [3], bereavement [4], cancer [5], human immunodeficiency virus (HIV) [6], suicide [7], assault [8], refugee [9], and combat veterans [10], and so on.

Furthermore, people with a strong connection to trauma victims, such as health personnel, family members, caregivers, social workers, and psychotherapists, have also demonstrated vicarious posttraumatic growth (VPTG) in the context of secondary trauma or alternative trauma [11]. It is particularly prevalent among professionals working with trauma survivors. They may experience personal and professional growth due to witnessing their clients’ resilience and ability to overcome adversity. These experiences include positive changes in self-cognition, interpersonal relationships, life values, increased compassion, sensitivity, and insight [12], and extraordinary growth in the context of one’s professional identity, which is professional’s job satisfaction and self-competence by witnessing the growth of their clients [13, 14].

The novel coronavirus (COVID-19) pandemic has had a profound global impact since its discovery in December 2019. As of March 11, 2023, the World Health Organization (WHO) has reported that the cumulative global cases of COVID-19 have surpassed 759 million, with nearly 6.9 million deaths [15]. Despite the WHO’s announcement on May 5, 2023, that the COVID-19 pandemic will no longer be classified as a global public health emergency, it is important to note that the threat to global health has not been eradicated. With the lifting of the state of emergency, it is imperative to address the psychological ramifications stemming from the pandemic [16]. The global healthcare system has been strained by the pandemic, placing a significant burden on healthcare workers (HCWs), particularly those in direct contact with COVID-19-diagnosed patients. This has resulted in a range of mental health issues, including pain, anxiety, burnout, depression, insomnia, posttraumatic stress disorder (PTSD), denial, and fear, which have adversely affected medical personnel, regardless of their direct or indirect exposure to trauma [17]. Despite the difficulties encountered, healthcare professionals can endeavor to adapt to demanding circumstances and rebound from traumatic experiences, which may result in favorable outcomes such as posttraumatic growth (PTG) or vicarious posttraumatic growth (VPTG). This inherent resilience among medical personnel is underpinned by their specialized training and extensive experience in crisis management, enabling them to navigate the uncertainties and pressures associated with the COVID-19 pandemic [18]. Furthermore, the daily exposure to life-and-death situations, coupled with a strong sense of professional duty, equips healthcare workers with the capacity to maintain composure and professionalism even in the face of overwhelming challenges [19]. These qualities not only facilitate their ability to cope but also lay the foundation for potential positive psychological outcomes such as PTG. Therefore, the exploration of PTG becomes integral in understanding how healthcare professionals not only withstand the adversities brought about by the pandemic but also use these experiences as catalysts for personal and professional development. The study of PTG is crucial not just for mitigating the negative psychological consequences of trauma but for promoting a resilient healthcare workforce, capable of not only enduring but thriving in the aftermath of significant challenges.

To the best of our knowledge, existing research on the level of PTG and its influencing factors among medical staff has presented some variability across different regions during the COVID-19 pandemic. A preliminary search for existing scoping reviews in systematic review databases, such as JBI, Cochrane, TRIP database, and PROSPERO, on 01 October 2022, revealed no systematic reviews or scoping reviews on this topic or any currently in progress. Consequently, this study, grounded in the scoping review methodology of Arksey and O’Malley [20], aims to (a) map the prevalence and characteristics of PTG in healthcare settings during the COVID-19 pandemic (b) identify key factors that may influence its development and (c) highlight the knowledge gaps for future research and interventions aimed at enhancing the psychological resilience and well-being of healthcare workers in the face of public health crises.

## Methods

This scoping review followed the scoping review framework developed by Arksey and O’Malley [20], including five stages: (1) identifying the research questions; (2) identifying relevant studies; (3) study selection; (4) charting the data; and (5) collating, summarizing and reporting the results. The results were based on the Preferred Reporting Items for Systematic Reviews and Meta-Analysis Extension for Scoping Reviews (PRISMA-ScR) checklist [21]. The PRISMA-ScR was intended to guide the reporting of this scoping review based on the relevance, credibility, and contribution of evidence. The completed PRISMA-ScR checklist can be found in Additional file 1.

### Search strategy

The literature was systematically reviewed between 01 January 2020 and 31 December 2022 using the following databases: PubMed, Web of Science, Embase, CINAHL, PsycINFO, China National Knowledge Infrastructure (CNKI), Chinese Biomedical Literature Service System (SinoMed), and Wanfang Data. A search strategy was developed by combining key and MESH terms by two research team members. The third member approved it of the research team and finally confirmed it by consulting the medical librarian. Here are the specific details: “COVID-19“[Mesh], “SARS-CoV-2“[Mesh], “COVID-19”, “SARS-CoV-2”, “coronavirus disease 2019”, “2019n-cov”; “Posttraumatic Growth, Psychological“[Mesh], “posttraumatic growth”, “post-traumatic growth”, “vicarious posttraumatic growth”, “secondary posttraumatic growth”, “alternative posttraumatic growth”; “Medical Staff“[Mesh], “Health Personnel“[Mesh], “Healthcare workers”, “health care provider”, “front line workers”, “medical workers”, “medical staff”, “healthcare professionals”, “nurse*”, “doctor*”, “physician”, “paramedic”, and adjusted in each database. The medrxiv.org and the references cited by the retrieved articles were also searched for additional references. The search strategies are detailed in Additional file 2.

### Study selection and eligibility criteria

Inclusion criteria were as follows: (1) the population described in the literature consisted of HCWs, including doctors, nurses, and other medical personnel who were directly or indirectly involved in the diagnosis, treatment, or care of patients with confirmed or suspected cases of COVID-19; (2) the research topic was “posttraumatic growth” or “alternative posttraumatic growth” associated with the COVID-19 outbreak and pandemic; (3) the situation and influencing factors of PTG were discussed in the literature; and (4) the study types included qualitative, quantitative, and mixed methods studies.

Exclusion criteria were as follows: (1) participants were medical students in clinical placement; (2) the study type was an intervention study; (3) the literature types were research protocol, conference literature, case reports, reviews, official reports, book reviews, letters to the editor, editorials, and studies published in preprint servers but not in peer-reviewed journals; and (4) duplicate and unavailable full-text, non-Chinese and non-English literature.

### Data extraction

The retrieved literature was imported into the NoteExpress software, and duplicates were checked. The title and abstract of citations were independently reviewed by two reviewers for the first screening level to identify articles that met the minimum inclusion criteria. A subsequent review of the full-text articles was conducted by two reviewers for the second screening level. Studies were excluded if they did not meet the eligibility criteria or were unrelated to the research question and purpose. Disagreements were discussed with a third researcher and resolved by consensus if there were any disagreements and uncertainties relating to study selection.

A form was developed to confirm the relevance and extract study characteristics from the included sources of evidence. Then, two reviewers independently charted the extracted data from each eligible article, and disagreements among reviewers were discussed. This process aimed to create a descriptive summary of the results, including aims, study design, nation, participants, tools/method, and main findings.

Evidence was combined qualitatively and descriptively after collecting the relevant data. The similarities and differences between the included studies, their relationship, situation, and risk factors of PTG were reported.

### Data integration methodology

This study employed a rigorous data integration approach to systematically synthesize and compare the results obtained from quantitative and qualitative research. This integration process aimed to gain a comprehensive understanding of PTG among medical staff during the COVID-19 pandemic. The following steps were adopted: (1) Identification of Common Themes: A detailed analysis of quantitative studies was conducted to identify key themes and patterns related to the characteristics and influencing factors of PTG. Concurrently, qualitative studies were thoroughly reviewed, employing content analysis to extract detailed individual experiences and nuanced insights on PTG. (2) Comprehensive Comparison: A comprehensive comparison table was established, encompassing the key statistical outcomes from quantitative research and the main themes from qualitative research. This table visually highlighted the consistencies and differences between the quantitative and qualitative research in terms of PTG characteristics and influencing factors. (3) Synthesis of Research Findings: The common themes from both research methodologies were synthesized. This included combining trends from quantitative data with profound insights from qualitative data to reveal a more comprehensive picture of PTG. Additionally, a detailed comparison and discussion were conducted to understand the perspectives and experiences captured by each research method in terms of PTG features and influencing factors. (4) Verification of Integrated Results: To ensure the accuracy and reliability of the integration process, the synthesized results were subjected to rigorous review by multiple researchers.

## Results

### Search results

**Fig. 1:**
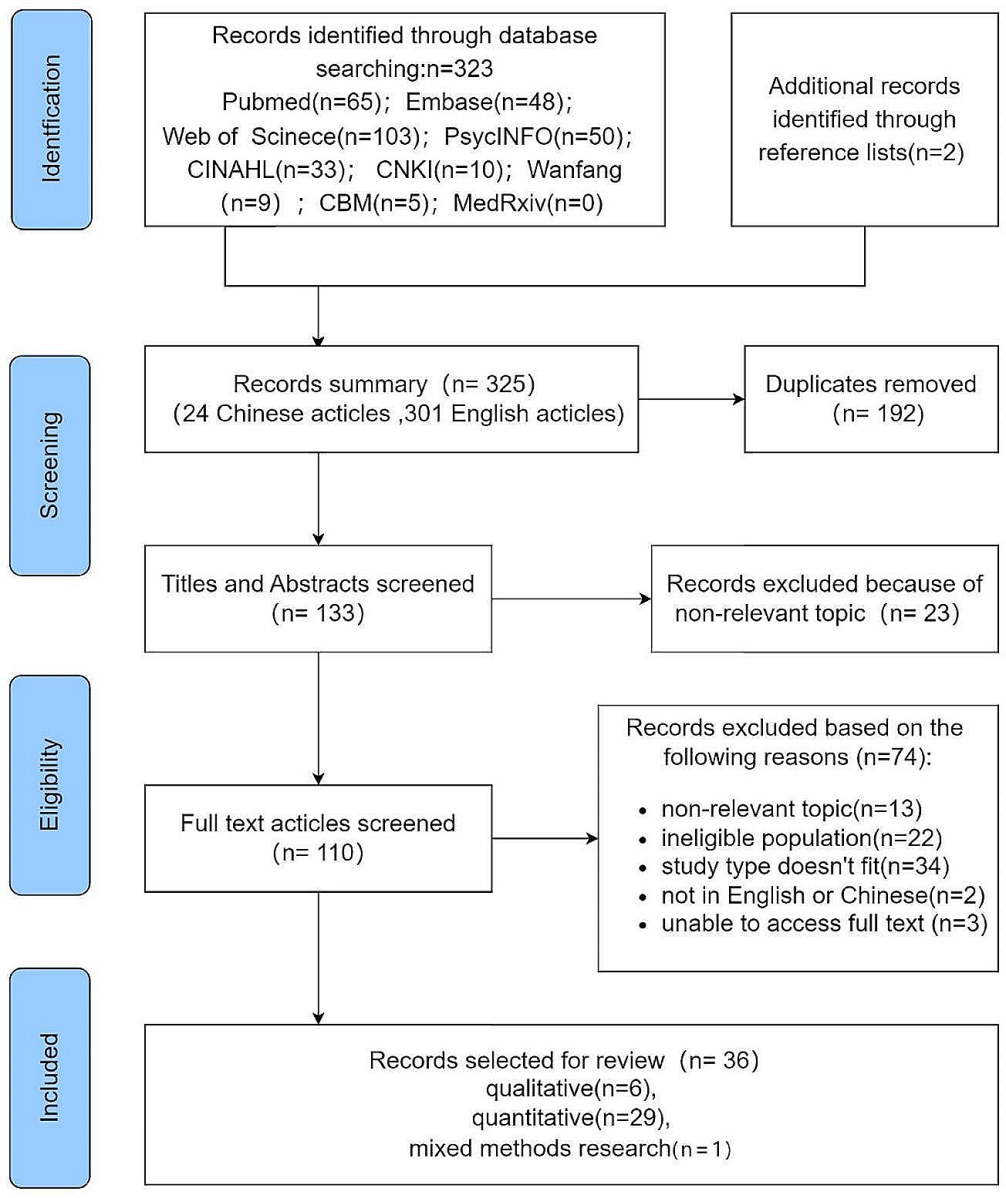
Flow diagram of screening of articles based on identified criteria

The primary search discovered 325 papers, including 24 Chinese and 301 English articles. After removing duplicates, 133 papers were left. We selected 110 articles for full-text reading based on screening titles and abstracts. We excluded 74 articles due to unrelated topics (n = 13), ineligible study populations (n = 22), noncompliant study designs (n = 34), not in English or Chinese (n = 2), and unobtainable full texts (n = 3). Finally, our analysis included 36 studies. Figure 1 presents a flowchart of the search strategy and the selection process based on identified criteria.

### Characteristics of sources of evidence

Five of the 36 articles were in Chinese, with the remaining 31 in English. Three were published in 2020, 15 in 2021, and 18 in 2022 (one of the studies was online in 2022 and published in 2023). The study included18 articles from China, four from Turkey, three from the United States, three from Korea, one from other countries, such as Greece, Italy, Canada, Spain, Israel, Serbia, and Palestine, and one from a global study covering three countries (Israel, Canada, and France).

The study population included 17 papers on frontline medical staff (care for COVID-19-diagnosed patients), four papers on nurses diagnosed with COVID-19 infection, and 15 on medical workers. It involves the emergency department, intensive care unit, dentistry, psychiatric department, and outpatient department. The medical institutions involved hospitals designated to treat patients diagnosed with COVID-19, general hospitals, communities, clinics, and other hospitals. The PTG level was measured at one to three-time points in 23 cross-sectional and six longitudinal studies, primarily using online questionnaires. Six qualitative articles utilized semi-structured interviews and questionnaires via telephone, video, or face-to-face interviews. Scale and open-question surveys were used in one mixed mothed record. Table 1 represents the general characteristics of the included literature.

**Table 1:** Overview of study characteristics included in the review

Study	Aims	Study design	Nation	Participants	Tools/Method	Main findings on PTG
Zhang et al. [22]	Explore trajectories and their predictors of PTG among frontline HCWs.	A longitudinal prospective cohort study: a three-wave, 2-year follow-up	China	565 frontline doctors and nurses	PTGI of Chinese Version	1) The PTG of HCWs with different characteristics showed different trends over time. 2) It is necessary to increase the measured frequency to understand the PTG status at different times. 3) Improving HCW’s resilience could help improve staff PTG.
Kapur et al. [23]	Assess PTG among radiation medicine staff members and changes in perceptions of departmental culture after the COVID-19 pandemic.	A longitudinal study: two-time points	America	213 members of the multicenter radiation department	PTGI	A fair-to-moderate degree of PTG was observed in personal and interpersonal relationship factors whereas the least growth was noted in spiritual and religious beliefs.
Yılmaz-Karaman et al. [24]	Evaluate the traumatic stress, anxiety, and depression levels of HCWs and their PTG levels during the pandemic.	A longitudinal study: two-time points	Turkey	66 HCWs cared for patients with COVID-19	PTGI	1) No significant differences appeared between the baseline scores and 6-month follow-up in the depression, anxiety, and traumatic stress levels of HCWs. 2) The PTGI scores decreased significantly over time. The depression and posttraumatic stress scores increased over time. 3) As the exposure to the stressors continues, individual traumatic stress levels increase, psychiatric disorders become frequent, and affirmative changes (like PTG) decline.
Zhang et al. [25]	Investigate how stress mindset and engagement in proactive coping behaviors predicted PTG among healthcare professionals during the COVID-19 pandemic in China	Cross-section design	China	589 health care professionals	PTGI-X	A stress-is-enhancing mindset predicts a higher PTG level among healthcare professionals during the COVID-19 pandemic in China, and the effect was mediated by engagement in proactive coping behaviors.
Atay et al. [26]	It aimed to determine PTG and psychological resilience and understand the relationship between PTG and psychological stability in frontline nurses.	Cross-section design	Turkey	263 nurses working at the pandemic clinics	PTGI	Psychological resilience was positively correlated with PTG and its subscales.
Jiang et al. [27]	Explore the process and influencing factors of PTG among emergency nurses infected with COVID-19.	Qualitative design based on the phenomenological approach	China	13 first-line emergency nurses infected with COVID-19	Semi-structured face-to-face interview	1) First-line emergency nurses infected with COVID-19 are a sensitive group that should be given more attention. 2) Investigating how they achieve psychological adjustment and growth in the case of severe trauma can provide valuable references for nursing management and education in the future. 3) Society, hospitals, and nursing managers should pay more attention to the PTG of nurses and establish supportive PTG strategies, which will benefit the retention rate and career development of nurses.
Veronese et al. [28]	Examine the relationship between stress of COVID-19 disease, psychological trauma, and burnout, and whether subjective well-being (SWB), sense of coherence (SOC), and PTG mediated the relationship between the three constructs in a group of HCWs.	Cross-section design	Palestinian	441 HCWs	Posttraumatic Growth Inventory-Short Form (PTGI-SF)	1) SWB, SOC, and PTG mediated the association between the stress of COVID-19, symptoms of trauma, and burnout. 2) SOC associated with SWB and PTG might be a protective factor against trauma during the pandemic.
Li et al. [29]	Assess Chinese nurses’ and the general public’ PTG during the COVID-19 pandemic.	Cross-sectional survey design	China	455 nurses (178 front-line nurses (FLNs) and 277 non-front-line nurses (nFLNs))	PTGI	1) The scores of total PTGI and all domains were significantly different between 178 frontline nurses (FLNs) and 277 non-front-line nurses (nFLNs). 2) Marriage status and ways to cope with stress were the predictors of PTG in nurses. 3) WeChat network psychological counseling and phone app application self-relaxation were excellent and effective coping strategies for nurses to relieve stress.
Yim et al. [30]	Establish a path model of PTG among nurses caring for COVID-19 patients and examine the associations between the relevant variables.	Cross-sectional study	Korea	229 nurses who cared for COVID-19 patients for more than one month	PTGI	1) Deliberate rumination directly influenced PTG and stress disorder, and social support, directly and indirectly, affected posttraumatic development. 2) To improve the PTG of nurses caring for COVID-19 patients, it is necessary to provide and support opportunities for self-disclosure.
Sarıalioğl et al. [31]	Examine the relationship between the transformative power of pain and PTG in nurses with positive Covid-19 PCR tests.	Cross-sectional study	Turkey	175 nurses with a positive Covid-19 PCR test	PTGI	PTG increased as the level of the transformative power of pain increased for nurses.
Yeung et al. [32]	Examined how sociodemographic characteristics, COVID-19-related worries, and work-related variables (satisfaction with work and workplace pandemic guidelines) were associated with PTG among nurses.	Cross-sectional study	China	1510 nurses working in hospitals and community	PTGI-SF	1) Nurses in Hong Kong did report positive changes amid the COVID-19 pandemic. 2) That working non-full-time, affiliating with a religion, having higher COVID-19-related worries and psychological distress, and having higher work satisfaction were associated with higher PTG. 3) Guideline satisfaction was only associated with higher PTG among those with higher distress but not those with lower pain.
Dahan et al. [33]	Examine personal levels of anxiety and concern, personal and national resilience (NR), and PTG.	Cross-section design	Israeli	183 mental health nurses	PTGI	1) A significant positive correlation was found between personal and NR and PTG. 2) Higher religiosity was associated with higher resilience, and higher professional seniority was related to higher PTG.
Kalaitzaki et al. [34]	Examine the associations among secondary traumatic stress symptoms(STS), VPTG, and coping strategies among HCWs during the COVID-19 lockdown and the mediating role of coping strategies in the STS-VPTG relationship.	Cross-section design	Greece	675 HCWs (41%physicians,37% nurses,12% social workers, and 10%psychologists)	PTGI	1) HCWs reported moderate to low STS and VPTG, with the VPTG dimensions of personal strength and appreciation of life being the highest categories. 2) Intrusions, mental, and both adaptive and maladaptive coping strategies predicted VPTG. 3) Adaptive coping strategies partially mediated the relationship between STS and VPTG, whereas maladaptive coping strategies fully mediated this relationship.
Mohamme et al. [35]	Explored the lived experience of Canadian frontline medicine nurses caring for COVID-19 patients during the first wave of the pandemic.	Qualitative research	Canada	43 frontline medicine nurses caring for COVID-19 patients	Semi-structured interviews and online surveys	Three overarching themes were deduced: (1) a traumatic experience, (2) living through the experience, and (3) achieving transcendence.
Feingold et al. [36]	Explore the prevalence, determinants, or correlates of PTG in frontline health care workers (FHCWs).	A prospective cohort: two-wave	America	787 HCWs are most likely to be directly involved in caring for patients infected with COVID-19.	PTGI-SF	1) A total of 76.8% of FHCWs endorsed moderate or greater PTG; the most prevalent domains were increased appreciation of life (67.0%), improved relationships (48.7%), and greater personal strength (44.1%). 2) Non-White race/ethnicity, more powerful positive emotions, pandemic-related PTSD symptoms, dispositional gratitude, and feelings of inspiration were independently associated with PTG.
Chen et al. [37]	Assess trauma, burnout, PTG, and associated factors for nurses in the COVID-19 pandemic.	Cross-section design	China	12,596 nurses	PTGI-SF	1) 13.3% reported trauma (Trauma ≥ 6), there were moderate degrees of emotional exhaustion, and 4,949 (39.3%) experienced PTG. 2) Nurses who identified as women working in ICUs, COVID-19-designated hospitals, and departments involved with treating COVID-19 patients had higher scores in mental health outcomes.
Cui et al. [38]	Explore the level and influencing factors of frontline nurses’ PTG during the COVID-19 epidemic	Cross-section design	China	167 frontline nurses	PTGI of Chinese Version	1) The PTG of frontline nurses was at a medium to high level. 2) PTG was influenced by working years, self-confidence in frontline work, awareness of risk, psychological intervention or training, and deliberate rumination. 3) It is necessary to strengthen psychological guidance and training for frontline nurses and promote their deliberate rumination on epidemic events to improve their PTG.
Peng et al. [39]	Assess PTG level and explore its influence factors among frontline nurses during the COVID-19 pandemic.	Cross-section design	China	116 frontline nurses	PTGI of Chinese Version	1) Moderate PGT was observed in the frontline nurses who had battled against COVID-19. 2) PTGI scores were significantly increased with family support, psychological comfort, and having children. The three factors only explained a 3.8% variance.
Aydin et al. [40]	Examined the experiences of nurses diagnosed with COVID-19 under the guidance of Meleis’ Transitions Theory.	Qualitative research	Turkey	18 nurses diagnosed with COVID-19	Semi-structured interview based on Meleis’ Transitions Theory concepts	1) The six themes that emerged in the data analysis were emotions experienced when nurses tested positive for COVID-19, emotions experienced during the quarantine process, PTG, methods of coping with COVID-19, nursing care after COVID-19 treatment, and metaphors about COVID-19. 2) Being diagnosed with COVID-19 caused nurses to have positive (PTG, empathic, and psychosocial nursing care) and negative experiences (fear of death, stigma, etc.). 3) They tried to cope with adverse situations due to COVID-19 by obtaining social support, thinking positively, and engaging in domestic activities.
Nie et al. [41]	To explore the relationships between relational capital, psychological security, PTG, and the meaning of work.	A cross-sectional questionnaire survey	China	760 frontline medical staff	PTGI of Chinese Version	1) Trust, reciprocity, and identification can promote PTG by enhancing the individual’s psychological security. 2) Work meaning has a moderated mediating effect when trust and reciprocity affect PTG through psychological security. Still, no mediated mediating impact is found when identification affects PTG through psychological safety.
Lyu et al. [42]	To explore the longitudinal relationship between resilience and PTG, as well as the role of job burnout in this relationship.	Longitudinal prospective cohort study: a three-wave in 2020	China	134 frontline HCWs from COVID-19‐designated hospital	PTGI of Chinese Version	1) Resilience at Time 1 positively predicted PTG at Time 2, which positively predicted stability at Time 3. PTG at Time 1 also positively predicted resilience at Time 2. 2) Job burnout was negatively related to both resilience and PTG; in particular, emotional exhaustion moderated the link between PTG and resilience. 3) There was a cycle of reinforcement between resilience and PTG over time. The positive effect of PTG on resilience, however, is undermined by emotional exhaustion.
Moreno-Jiménez et al. [43]	Examine the Secondary Traumatic Stress(STS)and PTG among healthcare professionals and the demands and resources related to COVID-19.	Longitudinal prospective cohort study: a two-wave in 2020	Spain	172 healthcare professionals from hospitals, healthcare centers, and nursing homes	PTGI-SF	1) Workload and fear of contagion in T2 were positive predictors for STS, whereas harmonious passion was a negative predictor. Both times, fear of contagion seemed to predict PTG and harmonious passion positively. 2) One moderation effect was found concerning the lack of staff/PPE, as PTG was higher when the workload was high, especially in those with an increased lack of staff/PPE.
Uziel et al. [44]	To study the attitudes, emotional responses, and worries among the dental personnel; and look for the ability of dental personnel to experience PTG due to the distress caused by the pandemic.	A cross-sectional online survey	Israel, Canada, and France	537 dental personnel(dental practitioners, dental hygienists, and dental assistants)	PTGI-SF	Responses of dental personnel to the COVID-19 pandemic varied worldwide. Despite the differences, evidence exists that some of the dental practitioners’ worries and concerns are associated with psychological growth as a result of the pandemic.
Mo et al. [45]	To investigate PTG and analyze its correlation with professional self-identity and social support.	A cross-sectional descriptive design	China	266 nurses who faced COVID-19 in Hubei Province	PTGI	1) Participants’ mean scores were 96.26 (SD = 21.57) for PTG. 2) Professional self-identity and social support were positively correlated with PTG. 3) Professional self-identity, social support, and whether nurses moved from other provinces to support Hubei Province were significant predictors of PTG.
Digiovanni et al. [46]	To examine the personal and professional impacts of the COVID-19 pandemic on the development, practice, and shifting values of child and adolescent psychiatrists (CAP) to inform how the field may move forward post-pandemic.	Qualitative research	America	24 child and adolescent psychiatrists	Semi-structured interviews	Three main thematic domains: (1) Unsettling, or “who have we been?” (identifying discontents such as daily inefficiencies and interprofessional loss of trust); (2) Adaptation, or “who are we now?” (exploring affordances and limitations of virtual work, and the evolution of personal and professional identity); and (3) Reimagination, or “who will we become?” (renewing a commitment to psychiatry as advocacy).
Zhang et al. [47]	To investigate the current status of PTG of clinical nurses and analyze it’s influencing factors.	A cross-sectional study	China	1790 nurses	PTGI	1) The total score of PTG of 1790 nurses was 67.17 ± 14.79. 2) Good social support and self-efficacy were essential factors in improving the level of PTG of clinical nurses. At the same time, a bad psychological state and working for many years were the negative factors of PTG.
Lee et al. [48]	To explore the experiences of COVID-19-designated hospital nurses in South Korea who provided care for patients based on their lived experiences.	Qualitative research	Korea	18 nurses working in a COVID-19-designated hospital	In-depth individual telephone interviews	Nine themes were identified: pushed onto the battlefield without any preparation, struggling on the frontline, altered daily life, low morale, unexpectedly long war, ambivalence toward patients, forces that keep me going, giving meaning to my work, and taking another step in one’s growth.
Carola et al. [49]	To examine the psychological effects on HCWs who cared for COVID-19 patients who were admitted to the intensive care unit in an Italian hospital.	Mixed research	Italy	35 COVID-19 intensive care unit HCWs	PTGI, Two open-ended questions	1) 50% showed PTG in the “appreciation of life” and “new possibilities” dimensions. 2) Three themes were identified: quality of workplace relationships, sense of emotional-relational competence, and sense of clinical-technical competence.
Li et al. [50]	To explore the mechanism of perceived organizational support on occupational resilience and PTG of nursing staff in the prevention and control of COVID-19.	A cross-sectional study	China	120 nurses working in Emergency, Infection, Fever Clinic, ICU	PTGI of Chinese Version	1) The score of PTG was (59.83 ± 11.20). 2) Career resilience, organizational support, and PTG were positively correlated. The mediating effect of perceived organizational support on career resilience and PTG was 0.15, accounting for 22.73% of the total effect.
Cui et al. [51]	To investigate the PTG and its related factors among nursing staff working against COVID-19 in the frontline clinical departments.	A cross-sectional study	China	512 nursing staff working in fever clinics and isolation wards during the COVID-19	PTGI of Chinese Version	1) The total score of PTGI was 64.35 ± 7.24. 2) The related factors of PTGI included professional titles, education, original working departments, time spent in frontline clinical departments, whether received training in disaster events, whether with similar working experience, whether relatives or colleagues being diagnosed with COVID-19, nurse-patient relationship satisfaction, whether with experience in the rescue of critically ill patients.
Li et al. [52]	To understand the PTG level and its influencing factors on nurses COVID-19 epidemic, and to provide a basis for the formulation of psychological crisis intervention programs in emergencies.	A cross-sectional study	China	382 outpatient and emergency nurses from COVID-19-designated hospitals.	PTGI	1) The total score of PTG was(74.30 ± 25.49). 2) The degree of concern about the epidemic prevention measures taken by the government, the degree of family care, and the degree of fear of infection of relatives and neighbors were the main factors influencing PTGI.
Lv et al. [53]	To explore the relationship between social support, resilience, expression suppression, and PTG among frontline medical workers.	A cross-sectional study	China	511 frontline medical workers from designated hospitals.	PTGI	1) Social support directly predicted PTG significantly and indirectly predicted PTG through resilience. 2) Expression suppression significantly moderated the relationship between social support and PTG.
Cai et al. [54]	To explore the experience of PTG of nurses with COVID-19.	Qualitative research	China	14 nurses designated with COVID-19.	Semi-structured in-depth interviews via video or telephone	The PTG of the nurses can be summarized into three categories: the emotional experience after COVID-19 infection, the PTG-promoting factors, and the PTG experience.
Han et al. [55]	This descriptive survey aimed to identify the factors affecting the PTG of nurses in COVID-19-designated hospitals based on a PTG model.	A cross-sectional study	Korea	233 nurses working at three COVID-19 hospitals	PTGI-X	The factors that significantly influenced the participants’ PTG were identified as marriage, religion, self-disclosure, deliberate rumination, meaning in life, and resilience.
Prekazi et al. [56]	To elucidate the positive impact of coping with the COVID-19 outbreak on mental health, such as PTG.	A cross-sectional study	Serbia	691 healthcare providers	PTGI	1) It showed low mean scores on PTG. Relating to Others was the highest scoring PTGI factor, with New Possibilities as second. 2) The effect of mental health on PTG was found to be mediated by coping skills. 3) Statistical differences across all PTG subscales except relation to others when comparing participants who experienced COVID-19 infection and those who did not.
Lv et al. [57]	To explore the specific relationships between family-work conflict and PTG as well as the specific roles of positive psychological capital, perceived social support, and suppression.	A cross-sectional study	China	1347 medical workers	PTGI	1) Positive psychological capital and perceived social support played mediating roles, while suppression strategies moderated the mediating effect. Compared with the low suppression group, the negative impact of family-work conflict on positive psychological capital and perceived social support was reduced in the high suppression group. 2) A higher level of suppression was more conducive to PTG.

### Synthesis of results

#### Quantitative results

**The level of posttraumatic growth**

The Posttraumatic Growth Inventory (PTGI), a 21-item scale developed by Tedeschi and Calhoun [2] in 1996, measures PTG, including five dimensions: relationship with others, new possibilities, personal strength, spiritual change, and appreciation of life. It is scored on a 6-point Likert scale from 0 to 5, ranging from “not at all” to “very much.” Researchers in various regions have modified this scale based on cultural adaptation. Most Chinese studies used the revised version of the 20-item PTGI by Wang Ji [58], deleting item 18, “My religious beliefs are stronger,” due to its low correlation with the total score and local culture in China. It adopted the Likert 6-point scale with a score of 0–100. Six studies [28, 32, 36, 37, 43, 44] used a 10-entry short version of the PTGI scale (PTGI-SF), adopting a Likert 6-point scale with a total score of 0 to 50 [59]. Tedeschi et al. [60] updated the list with four new items in the spiritual and existential change subscale to better capture spiritual and existential change in non-religious cultures, comprising PTGI-X with 25 items scored from 0 to 125 with a 6-point Likert scale. Two studies included in this review used it as a measurement [25, 55]. PTG levels reached moderate and above with mean item scores of PTGI > 3 or total scores > 60 in two studies [24, 38]. However, another study indicates that people who scored higher than the 60th percentile might have grown [37].

HCWs experienced varying PTG levels following direct or indirect trauma during the COVID-19 epidemic. A total of 28 studies in the included literature reported specific PTGI scores, with moderate PTG levels in general and high scores on the dimension of “relating to others”, “appreciation of life”, and “personal strength” more frequently mentioned. Table 2 presents the details.

**Table 2:** Posttraumatic growth level of healthcare workers

Tool	Study	PTG levels		
		Total score	Singel item mean score	The highest dimension score
PTGI, 21 items, individual item scores 0 to 5; total score 0-105, five dimensions	Li et al. [29]	63.28 ± 23.41	-	Relating to others
	Sarıalioğlu et al. [31]	50.98 ± 25.30	-	Relating to others
	Kalaitzaki et al. [34]	46.60 ± 24.61	-	Relating to others
	Mo et al. [45]	96.26 ± 21.57	-	Relating to others
	Zhang et al. [47]	67.17 ± 14.79	-	Relating to others
	Li et al. [52]	74.30 ± 25.49	-	Appreciation of life
	Lv et al. [53]	-	3.36 ± 0.72	-
	Yılmaz-Karaman et al. [24]	T1:45.04 ± 26.39 T2:37.89 ± 26.28	-	-
	Kapur et al. [23]	T1:47.7 ± 28.3 T2:46.7 ± 28.2	-	Personal strength
	Prekazi et al. [56]	Mean scores: 47.13	-	Relating to others
	Lv et al. [57]	81.81 ± 19.547	-	-
Turkish version of PTGI, 21 items, single item scores 0 to 5; total score 0-105, three dimensions	Atay et al. [26]	69.95 ± 15.73	-	Self-perception
The Hebrew version of PTGI, 21 items, single item scores 1 to 4	Dahan et al. [33]	-	3.01 ± 0.81	-
The Chinese version of PTGI, 20 items, individual item scores 0 to 5; total score 0-100.	Li et al. [50]	59.83 ± 11.20	-	New possibilities
	Cui et al. [38]	70.53 ± 17.26	3.38 ± 1.21	Appreciation of life
	Nie et al. [41]	-	3.98 ± 0.723	-
	Cui et al. [51]	64.35 ± 7.24	-	Relationships and personal development
	Peng et al. [39]	65.65 ± 11.50	3.28 ± 0.57	Appreciation of life
	Lyu et al. [42]	-	T1:3.43 ± 0.66 T2(1 M): 3.92 ± 0.70 T3(3 M):2.90 ± 0.64	-
	Zhang et al. [22]	-	T(2020.1):2.89 ± 1.14 T(2021.5):3.04 ± 0.92 T(2022.3):3.40 ± 0.80	-
PTGI-SF, ten items, single item scores 0 to 5; total score 0–50.	Veronese et al. [28]	-	3.62 ± 0.90	-
	Chen et al. [37]	28.0 ± 11.54	-	Relating to others
	Moreno-Jiménez et al. [43]	-	4.11 ± 0.84	-
	Yeung et al. [32]	-	2.19 ± 0.97	-
	Uziel et al. [44]	Israel:17.83 ± 10.30 France:20.50 ± 11.13 Canada:21.43 ± 12.17	-	-
PTGI-X,25 items, single item scores 0 to 5; total score 0-125.	Zhang et al. [25]	76.74 ± 27.13	-	Personal strength
	Han et al. [55]	60.15 ± 24.59	2.41 ± 0.98	New possibilities
Korea version of PTGI, 16 items, single item scores 0 to 5; total score 0–80.	Yim et al. [30]	43.80 ± 14.65	-	-

“-” means not mention

**The influencing factors of posttraumatic growth**

**Trauma**

COVID-19 could be categorized as a new type of mass trauma. Different types of trauma-related scenarios or characteristics may influence PTG levels. COVID-19-exposed HCWs, such as those working in intensive care units (ICUs) or frontline or sentinel hospitals where confirmed cases are treated, had higher PTG levels [23, 29, 37, 45, 51]. HCWs diagnosed with COVID-19 or have a family member, friend, or colleague who has been diagnosed had higher PTG levels [51, 56].

The PTG levels of HCWs differed at different stages of the COVID-19 pandemic. A longitudinal study of frontline HCWs (n = 134) in China showed that Time 1 (Feb 2020) to Time 2 (Mar 2020) participants revealed an increase in PTG, while Time 3 (May 2020) participants indicated a decrease in PTG [42]. Another three-wave longitudinal study (n = 565) from China discovered that PTG gradually increased over two years of follow-up among HCWs, and four types of PTG trajectory were identified: persistent, steady increase, high with a drop, and fluid trajectory [22]. However, one study (two-point survey) from Turkey indicated that PTGI scores decreased significantly over time among 66 HCWs participated in the study [24]. Another two-wave survey from the U.S. radiology staff revealed a consistent trend toward lower PTGI [23].

The severity of the traumatic event and PTG in HCWs may have a positive or negative correlation. Researchers have also discovered that PTG was negatively correlated with trauma [28] and PTSD symptoms [47]. Another study indicated that PTG was positively correlated with PTSD [36].

**Demographic characteristics**

Gender, age, work years, job title, education level, marital status, child status, religion, and race may be associated with PTG. Most studies discovered higher PTG levels among HCWs with older age [22, 23], longer working years [33, 38], higher job titles [51], and higher education levels [22, 51]. However, some studies revealed that PTG was negatively correlated with age [25, 34] and professional title [47]. Not coincidentally, gender differences were also observed across studies. A survey of 455 nurses from China indicated that women had lower PTGI scores than men [29]. A survey of 673 HCWs from Greece showed that women scored higher on all VPTG subscales [34]. However, another large (n = 12,596) study from China demonstrated a greater trauma response in women than in men but no difference in PTG [37]. Similarly, a study from Serbia produced consistent results [56].

Additionally, HCWs with religious beliefs [32, 33], married [29], with children [39], and working part-time [32] had higher PTG levels. PTG levels differed between physicians and nurse assistants [43], and whether they were white [36] or born locally also differed from PTG levels [33]. Disaster training, rescue, critical patient resuscitation, and infectious disease treatment experience contribute to a higher PTG level [51].

**Psychological factors or personal traits**

Positive emotions or psychology or personal traits can promote PTG, such as resilience [22, 26, 33, 42], occupational resilience [50], occupational identity [45], self-efficacy [47], deliberate rumination [30, 38, 55], subjective well-being [28], coherence [28], harmonious passion [43], frontline job confidence [38], risk awareness [38], transformative power of pain [31], trust, reciprocity, and identification [41], being psychological comfort [39], and positive emotions and dispositional gratitude [36], as mentioned in most studies.

Negative emotions or psychological or personal traits can inhibit the PTG onset; examples include COVID-19-related stress/anxiety/concern [28] and job burnout [28, 42]. However, similar to trauma, the stress/anxiety/concern associated with COVID-19 has also revealed a double-edged sword effect on PTG. For instance, research from China exhibited that higher COVID-19-related worries and psychological distress meant a higher PTG level [32]. Studies from other regions have demonstrated the same effect [43, 44]. An increased stress mindset, determining the stress response, is associated with higher PTG levels [25].

**Coping and social support**

A positive coping style can contribute to PTG, including conducting psychological interventions/training, engaging in online counseling, and phone app of application self-relaxation [25, 29, 38, 47, 56].

A positive association has been demonstrated between PTG and social support, including support from organizations [26, 50], societies [30, 45, 47, 53], families [39, 52], and friends [39]. Additionally, good working relationships, such as nurse-patient satisfaction [51] and job satisfaction [32], can promote PTG in HCWs.

Seven studies explored the path analysis of the PTG influencing factors and discovered the mediating and moderating factors under their respective theoretical models, such as organizational support [50], social support [30, 57], coping strategies [25, 34], resilience [53], psychological security [41], expressive suppression [53], deliberate rumination [30], emotional exhaustion [42], self-disclosure [30], and positive psychological capital [57].

#### Qualitative results

Six qualitative studies [27, 35, 40, 46, 48, 54] described the specific experiences of HCWs when confronted with or diagnosed with COVID-19 through three periods of stress/negativity, adjustment to adaptation, and growth, presenting PTG occurrence. The qualitative part of another mixed study [49] identified three themes: quality of workplace relationships, sense of emotional-relational competence, and clinical-technical competence. Each theme has two broad macro categories: growth and block.

**Change in relationships with others**

Six of the Seven studies contributed to this theme. Improved interpersonal relationships include with family, friends, colleagues, and patients. Family, friends, and colleagues’ warm love and support bring their relationship closer and more intimate. A nurse diagnosed with COVID-19 remarked, “My boyfriend cared for me, encouraged me, and gave me strength after I got sick; I will cherish the relationship between us” [54]. “When I saw my son at the gate of the community after I came back from isolation, I burst into tears and held him tightly in my arms” [27]. As healthcare worker spends more time with a patient, their empathy and compassion for the patient gradually intensifies. Like comrades who fought back the “enemy” (COVID-19), both sides cheered and encouraged each other to overcome difficulties and diseases, improving the relationship between doctors and patients. One nurse said, “When the test result returned negative for the first time after being admitted, I was so happy and cried together with the patients” [48]. Additionally, the experience of being a patient after a COVID-19 diagnosis also influences how HCWs treat patients, and role reversal and empathy improve the relationship with patients to some extent [27, 54]. During this particular time, colleagues’ help, care, and encouragement in caring for infected patients promote teamwork and interpersonal relationships [27, 35, 48, 49, 54].

**Increase in individual strength**

This resulted in a shift in participants’ mental and professional perceptions of themselves. At a psychological level, HCWs reported that the experience had made them more courageous, strong, and optimistic. “I think I am a little more brave and strong than I thought I would be” [54]. In the face of difficulties or trauma, resilience allows individuals to make positive choices and respond rationally to stress. This facilitates guiding individuals to reconstruct non-adaptive states and activate their potential to resist crises to resolve difficulties. Most HCWs described their experiences exploring and reconfiguring their strengths [27].

At the professional level, HCWs had a positive attitude toward gaining work experience related to a new infectious disease [48, 49]. They viewed their current experience as a valuable opportunity to learn new skills and enhance their work, gradually moving from unfamiliarity at the beginning to completing the work previously given to the nurse aides and being able to quickly shift and focus on enhancing the quality of care and improving patient well-being. This adds significance to their experience [48].

**Changes in the philosophy of life and priorities**

Four of the seven studies contributed to this theme. Interviewees mentioned a new appreciation of life and the future after experiencing trauma. They will re-examine life’s meaning and re-plan their future priorities, such as“I felt the need to live more meaningfully as the disease gave me another chance to live. I became more attached to life and realized how valuable it is …” [40].

Most life priorities change are reflected in the increased priority given to physical health. “Nothing is better than a healthy life, and nothing is as important as health” [27]. “… I realized that health is more important than anything else. Thus, I decided to stop worrying about some things, stop overthinking, and stop to give importance. I realized that health is the most important thing” [40]. Moreover, it is reflected in other meaningful and fun things, such as “I will get better for myself and my family. I will spend more time with them, cherish every day, and enjoy the fun of life. I still have many important tasks to complete” [27].

**Self-identification of profession**

Participants in four studies described greater vocational identity. Most participants expressed satisfaction and pride that they were making a concrete contribution to the fight against the global pandemic. Their pride was further enhanced with increased social recognition of HCWs caring for COVID-19 patients. All of these enhanced their professional identity. “The work that I am doing is truly helping others. I am contributing during this national disaster situation. I am here at this historical moment…” [48], “I am proud to be a nurse and to have assisted on the front lines” [35], and “I think every HCW is a hero” [54]. As child and adolescent psychiatrists, they have experienced a successful transition from “who we are” and “what we can do now” to “who we will become” during the pandemic and then engendered a reevaluation of and a recommitment to psychiatry [46].

**Spiritual change**

One research reported a change in spirituality [40]. After being diagnosed with COVID-19, the nurses questioned their spiritual lives and changed. “Inevitably, death anxiety enters your mind, and you question yourself. I realized how spiritually weak I was and made a promise to myself. I would pay more attention to my prayers after the treatment … I was angry with myself as I was living in this way….” “Thus, I realized that everything was in vain; the only real thing is after death. … I started to question my mistakes and sins and plan to get rid of them … I turned to God more.” Many of them rely on religious beliefs to manage stress.

#### Integration of quantitative and qualitative research

To provide a clearer understanding of the consistency and divergence between quantitative and qualitative studies in the PTG of HCWs, we have established Table 3 to compare the associations of these two research methods regarding PTG characteristics and influencing factors.

**Table 3:** Comparison of PTG characteristics and influencing factors in quantitative and qualitative results

Items	Themes	Qualitative results	Quantitative results
Characteristics	Relationships with others	Valuing harmonious interpersonal relationships Closer relationships with family, friends, and colleagues, willing to help Gratitude for colleagues Improve relationships with patients Enhancing empathy and humanistic care	The versions and scores of PTGI vary across cultures The criteria for classifying different PTG levels varied across studies Overall medical staff scored moderate on the PTGI Higher scores on the dimensions of “relationships with others”, “appreciation of life” and “personal strength”.
	Appreciation of life	Builds a new outlook on life, realizes the value of life Re-examine life’s meaning, re-plan future priorities Giving meaning to my work The most important thing is health Recommitment to the profession and its purpose Seeking collaboration and reinvention Be grateful	
	Personal strength	Discovering a better self Stimulates the potential of personal strengths, forces that keep me going Identifying with one’s profession, feeling proud of nursing Accomplishment Expecting to return, fighting together with fellow nurses Providing real nursing Confidence in caring for infected patients Broadening perspectives	
	New possibilities	Finding a new direction Self-transformation	
	Spirituality change	Pay more attention to prayers Religious beliefs	
Influencing factors	Work-related stressors	Changes in practice: team, knowledge, skills Concerns for safety Communication challenges	Trauma: trauma-related scenarios or characteristics, during different stages of the COVID-19 pandemic, trauma severity.
	Psychological stress and emotional reactions	Fear and anxiety Helplessness and anger Escapism Frustration and depressed mood Ambivalence toward patients Low morale	
	Psychological factors or personal traits	Psychological capital Resilience Positive Thinking	Positive emotions and psychological traits can promote PTG (resilience, occupational identity, self-efficacy, deliberate rumination, etc.), while negative ones can inhibit the PTG onset (stress, anxiety, concern, job burnout, etc.).
	Coping and social support	Effective coping (self-motivation, avoidance, and distraction) Adequate support network (support from the community and organizations, healthcare professionals, family and friends)	Positive coping style (psychological interventions/training/counseling, self-relaxation); Support from the organizations, healthcare professionals, family, and friends.
	Profession and identity	Adaptation to new roles and identities (adapting to virtual work and the evolution of personal and professional identities) Giving meaning to my work (perceived professional calling, pride in serving COVID-19 patients)	Demographic characteristics: Gender, age, work years, job title, education level, marital status, child status, religion, race, disaster training, rescue, critical patient resuscitation, and infectious disease treatment experience.

Through the comparison presented in the table, we observe a notable coherence and complementarity in understanding the characteristics and influencing factors of PTG among HCWs during the COVID-19 pandemic. In terms of PTG characteristics, the themes distilled from qualitative studies correspond closely with the five dimensions measured in quantitative PTG scales. This alignment elucidates the specific contexts and manifestations of these dimensions, providing a clearer and more comprehensive understanding of what PTG looks like for HCWs in the context of the pandemic. Regarding influencing factors, there is a synergistic relationship between the themes identified in qualitative research and the factors statistically derived from quantitative studies. For instance, qualitative themes such as “Work-related stressors” and “Psychological stress and emotional reactions” offer a vivid explanation of HCWs’ early responses to the “Trauma” of the COVID-19 pandemic. Additionally, qualitative findings explicate how internal and external factors foster PTG, detailing the process of its formation. This consistency and complementarity between qualitative and quantitative approaches highlight the importance and value of employing a combined methodological perspective for a holistic understanding of PTG.

## Discussion

This literature review aimed to provide insight into the existing evidence base of what PTG looks like in HCWs and internal and external factors that may contribute to and hinder this phenomenon.

### Summary of findings

Thirty-six papers from 12 countries met the inclusion criteria. In the context of the COVID-19 pandemic, HCWs faced tremendous stress and strain, generating associated mental health problems. They were also stimulated to adapt and adjust, generating PTG; the most notable included “interpersonal relationships,” “changes in life philosophy,” and “growth in personal power”. The factors influencing PTG were the level of trauma exposure, sociodemographics, and the psychological traits of the traumatized individual, such as psychological resilience, positive psychological qualities, and coping and social support.

### Integrated discussion

Our study reveals the alignment and complementarity between qualitative and quantitative research. The integration of these methodologies not only enriches our understanding of PTG’s features but also enhances our grasp of its complexity. The themes identified in qualitative research correspond to the dimensions of the PTGI used in quantitative studies, elucidating the specific contexts and manifestations of these dimensions within the healthcare environment. For example, the growth in the personal strength dimension of PTGI, when reflected in the workplace, manifests as enhanced skills, increased confidence, professional identity, and a sense of accomplishment [46, 48, 54]. Moreover, the detailed backgrounds and descriptions provided by qualitative research help explain how factors identified in quantitative studies facilitate PTG. For instance, quantitative data may show a correlation between social support and increased PTG, but qualitative insights explain the mechanisms and reasons behind these relationships [27, 48, 54].

Qualitative research, with its intricate depiction of the phenomenon, addresses the limitations of quantitative research, enriching our understanding across various dimensions. This profound understanding aids in a more nuanced grasp of the essence and complexity of posttraumatic growth. Ultimately, this integrative approach not only broadens our comprehension of PTG but also underscores the value of combining qualitative and quantitative methods in mental health research. It provides a blueprint for future studies, demonstrating the importance of methodological diversity for a comprehensive understanding of complex psychological phenomena.

### What does posttraumatic growth look like?

The variation in cultural backgrounds, measurement instruments, dimensions, and the reliability and validity of PTG assessments made it difficult to compare specific values across studies. Discrepancies in PTG levels might also be attributed to differences in survey time frames, health service contexts, pandemic control measures, and individuals’ subjective appraisals of COVID-19-related stressors. The criteria for classifying different PTG levels varied across studies. Several studies [24, 37, 38] used total scores > 60, single entry scores > 3, or ≥ 60% of the total scores as an intermediate or higher level of PTG, while others did not report delineation criteria. Overall, a medium level of PTG was observed. The COVID-19 pandemic has imposed enormous stress on HCWs, leading to mental health problems. Nevertheless, they adapt and adjust, ultimately yielding positive behaviors and experiences. The pandemic prompted the most profound changes in “human relationships,” “philosophy of life,” and “personal strength,” consistent with the qualitative studies review.

After experiencing an initial period of negative emotions in response to acute stress, individuals mobilize internal and external resources to manage the effects of trauma. Internal resources, such as mental toughness, intentional reflection, and resilience, with external resources like organizational, familial, and societal support, contribute to the positive outcome known as growth [61]. During this process, support from others facilitates adaptation and adjustment while manifesting in growth expressions, enhancing relationships, and creating reciprocal influence. After the trauma, individuals develop a positive and transcendent view of themselves and a new philosophy of life, appreciating various aspects, including health, existence, subtle experiences, and relationships.

### What affects posttraumatic growth?

#### Trauma

The COVID-19 pandemic, as a traumatic event, has disrupted the assumptive world of HCWs, leading to their dysregulated cognitions and emotions. However, according to the model of “Posttraumatic Growth at Work” [52], they may achieve PTG via a recursive cycle of emotion regulation and sensemaking.

First, individuals with high trauma levels of exposure may exhibit severe PTSD symptoms, but higher trauma levels of exposure do not necessarily imply higher growth levels. There is no PTG without trauma for individuals. However, mild trauma may be insufficient to stimulate growth, and severe trauma may be more detrimental than beneficial to growth [62]. Thus, only moderate trauma exposure may trigger individual PTG and provide room for growth, demonstrating a positive correlation. For instance, a study discovered a linear and curvilinear relationship between trauma and posttraumatic development, and moderate indirect trauma was associated with the highest PTG levels [63]. A network analysis of war-related PTG revealed a U-shaped relationship between posttraumatic stress symptoms (PTSS) and PTG levels [9]. Additional studies supported these findings. The work experience of inpatient psychiatric nurses was associated with higher PTSD levels and secondary trauma. However, their growth was significantly slower compared to community nurses, which were thought to be associated with higher and continuous exposure to trauma and fewer opportunities to take breaks to reflect on it [64].

Second, although traumatic events are transient, their effects are continuous and dynamic, and post-traumatic stress and growth levels can change over time. For instance, a longitudinal study of tsunami survivors discovered that PTG could moderate the relationship between posttraumatic stress, depression, and quality of life after natural disasters [65]. Another tracking survey of earthquake survivors revealed that growth could reduce long-term PTSS [66]. Survivors may undergo self-adjustment and receive external support or intervention during the post-trauma period, which can accumulate over time and lead to different levels of trauma and growth. Their relationship remains uncertain due to the complex symbiotic relationship between trauma and growth and the many factors involved [67, 68].

#### Demographic characteristics

Although there are differences in the relationship between age, years of work, job title, education level, marital status, parental status, whether they were born in the local area, and PTG, they all share common factors that influence their growth. HCWs with more internal and external coping resources and a greater ability to cope with trauma exhibit higher PTG levels. Higher age, higher job title, higher years of work experience, and higher education level mean more internal resources, such as work experience and life experience, allowing them to use their clinical skills, integrate external resources through reflection, and then adopt a positive coping approach when faced with an unexpected pandemic. However, some studies also proved a negative correlation between age and growth [25, 34]. It might result from young people’s greater willingness to change their cognitive patterns and derive positive meaning from trauma, consistent with previous research on age differences in PTG [69]. Being married and having children may provide more emotional and material support resources from the family system, leading to higher PTG levels. According to other studies, the relationship between gender and PTG differs between studies [29, 34, 37], possibly due to the influence of other factors, such as the gender interaction with pandemic duration and individual quarantine [70].

HCWs who have received disaster training, rescue experience, critical patient resuscitation experience, and infectious disease treatment experience exhibit greater composure when faced with COVID-19. They can utilize their professional skills effectively to provide better care for critically ill patients and even lead other HCWs to do the same. This strengthens their sense of professional identity and honor and is associated with higher PTG levels [51].

Additionally, religiously engaged healthcare professionals have been shown to effectively use their faith and spirituality to cope with adversity during disasters. Religious beliefs can provide a framework to positively view threatening situations, facilitating a sense of challenge and growth through suffering [71]. Previous research has also highlighted the social support function of religious involvement [72]. Part-time nurses have higher PTG levels than full-time nurses [32]. This could be due to the extra time part-time employees must devote to their nursing duties and stressful experiences, potentially contributing to PTG.

Demographic characteristics may be associated with an individual’s emotional regulation and psychological resources in response to traumatic events. When designing interventions, the influence of these sociodemographic characteristics on PTG should be considered. Understanding these features carries significant practical implications for promoting PTG.

#### Psychological factors or personal traits

Regarding the temporal course of post-traumatic experiences, it has been suggested that intrusive rumination in the early stages of trauma can exacerbate psychological distress, elicit negative emotions and outcomes, and hinder PTG. However, as time progresses, an individual’s positive psychological resources, such as positive worldviews (hope, tolerance, psychological resilience, optimism, self-esteem, wisdom, and spirituality), positive emotions (happiness, gratitude, and satisfaction), and positive attitudes towards society (social cohesion, altruistic behavior, social responsibility, and benevolence), can aid in overcoming negative emotions and enhancing coping abilities [73]. Intrusive rumination may give way to active reprocessing and constructive thinking about the traumatic event under the influence of positive self-adjustment and social support. Moreover, negative reviews in the early stages of trauma can provide material for subsequent positive processing, enabling individuals to discover the positive meaning of the traumatic experience, ultimately promoting growth.

Psychological resilience, also known as resilience, refers to the ability to adapt or recover from highly adverse circumstances. It is a source of strength that enables individuals to remain well-adjusted, develop, and grow, and a key factor influencing whether they can overcome difficulties and adversity. However, the relationship between resilience and PTG remains controversial. Previous studies have suggested that their relationship may be positive [74], curvilinear [75], or insignificant [76], and further investigation is needed to explore the mediating and moderating factors involved. Considering the dynamic nature of resilience and PTG over time, Lyu [42] explored their trends and relationships throughout the traumatic event and discovered that individuals continue to grow in a virtuous cycle after trauma. PTG promotes psychological resilience, and PTG promotes psychological resilience. Over time, these two factors positively influence each other, contributing to the individual’s continued positive functioning during and after the adverse experience.

Psychological resilience and positive psychological qualities can assist HCWs in better emotion regulation and sensemaking, thereby promoting PTG. This finding is consistent with the model of “Posttraumatic Growth at Work” [77], confirming the importance of these individual characteristics for PTG. Therefore, enhancing psychological resilience and positive psychological qualities in medical staff can improve their growth potential in the face of traumatic events.

#### Coping and social support

Effective coping styles and strong social support are vital in facilitating PTG. Positive and effective coping strategies help individuals face challenges and mobilize resources from others and society to solve problems. Psychological interventions or training during or before frontline work can help HCWs maintain positive emotions, reduce attentional bias towards negative emotions, and facilitate their regulation and release, enabling them to perform their high-intensity work in a good psychological state. These interventions or training may also stimulate HCWs’ sense of mission and professional responsibility, generate positive psychological experiences, and promote growth in their front-line work [38]. Excessive attention to negative external information can trigger negative emotions, but shifting attention appropriately can help traumatized individuals detach from distress and gain new insights to reexamine and confront traumatic events. Self-motivation can enhance an individual’s self-confidence and facilitate positive psychological adjustment, prompting the traumatized person to adopt positive behaviors, solve problems, and grow. Choosing a suitable relaxation method, such as online counseling or a self-relaxation mobile app, can also help to cope effectively with pandemic stress [29].

Social support refers to the material and emotional assistance individuals receive from their social networks, including family, friends, and other socially connected individuals. Individuals can process traumatic events in a supportive environment by disclosing their internal processes to others, particularly when focusing on cognitive and emotional factors. The degree to which individuals perceive their social environment as encouraging or inhibiting plays a crucial role in the PTG process. Adequate protective materials can help HCWs better protect themselves and increase their confidence in their work, while care and support from family, friends, colleagues, and social organizations can alleviate stress and anxiety during the COVID-19 pandemic [26, 30, 39, 45, 47, 52, 53]. Good social support provides HCWs with external resources and emotional support, creating a safe atmosphere for self-expression, understanding, and acceptance [30].

Effective coping strategies and robust social support significantly impact PTG, aligning with the emphasis on social support, occupational backing, and attentive companionship in the “Posttraumatic Growth at Work” model [77]. This support can assist HCWs in modulating their emotions and provide a safe environment for sensemaking, thereby facilitating PTG. It is crucial to strengthen the training of coping abilities for medical staff and elevate social support during intervention measures.

### Limitations and directions for future research

This study has several limitations that should be acknowledged. First, it only provided a descriptive analysis of the included literature and did not rigorously evaluate the studies’ quality. Second, the review focused exclusively on “posttraumatic growth” or “alternative posttraumatic growth,” omitting relevant topics such as positive posttraumatic experiences and perceived benefits. Third, only 28 of the 36 papers provided specific PTGI scores, and the data could not be integrated due to variations in PTGI versions, classification criteria, and result presentation. Additionally, most studies were cross-sectional, precluding the establishment of causal relationships. Some studies on risk factors did not control for confounding variables (work hours, COVID-19 exposure intensity, cultural background, and government policies), potentially affecting the results.

Our study’s findings point to several critical directions for future research to enhance the understanding of PTG among HCWs. Firstly, there is an urgent need for additional longitudinal studies to delve deeper into the dynamics and formation mechanisms of PTG. Such studies are essential for providing a more comprehensive understanding of how PTG evolves. Secondly, considering the global scope of the pandemic, it is crucial to understand PTG within various cultural contexts. Cultural differences in emotional experiences and expressions can significantly influence the process and potential of PTG. Therefore, future research should incorporate a cultural perspective, exploring how cultural factors impact the development and experience of PTG among healthcare professionals. This approach will not only enrich our understanding of PTG in diverse settings but also guide culturally sensitive support and intervention strategies. Lastly, future studies should focus on assessing various interventions’ efficacy to determine best practices for supporting HCWs’ psychological well-being and growth. This includes exploring how different types of support systems, both professional and societal, can facilitate PTG. The development and validation of these interventions will provide critical guidance for healthcare settings and policymakers in creating robust mechanisms to support HCWs during and after traumatic events.

### Implications for practice and policy

The psychological health and PTG of medical staff are long-term concerns. To enhance the psychological resilience and PTG levels in medical staff, practice and policy should focus on the following aspects:

In terms of practical implications, we underscore the necessity of enhancing support systems tailored to the specific needs of HCWs. This involves developing interventions that address the key factors influencing PTG, such as trauma exposure and coping strategies. Such support systems could encompass resilience training programs, mental health workshops, and peer support initiatives, all designed to fortify the psychological resilience of healthcare staff. Additionally, there is a need for personalized interventions that take into account the individual psychological traits and sociodemographic factors of HCWs, thereby fostering PTG in a manner that resonates with their unique experiences and backgrounds. Furthermore, it is important to update the existing crisis response protocols to integrate measures for psychological well-being. This would ensure that HCWs’ mental health is a primary consideration during pandemics, aligning crisis responses with the psychological needs and challenges faced by these essential personnel.

Regarding policy implications, our study highlights the critical need for strategic resource allocation to enhance mental health services and support systems in healthcare settings. This is particularly crucial during public health emergencies, such as the COVID-19 pandemic, where the mental health demands of HCWs are significantly heightened. Adequate resource allocation should include not only immediate support but also long-term mental health services to address the ongoing needs of healthcare professionals. Recognizing the lasting impact of pandemic experiences on HCWs, it is imperative to develop comprehensive long-term mental health strategies. These strategies should encompass continuous support, regular mental health assessments, and adaptive interventions, ensuring that the evolving mental health needs of HCWs are met effectively. Such policies would not only provide immediate relief during crises but also contribute to the sustainable well-being and resilience of healthcare professionals in the long run.

## Conclusions

This scoping review revealed that medical staff experienced moderate PTG during the COVID-19 pandemic, with notable improvements in interpersonal relationships, life philosophy, and personal competence. Key influencing factors included trauma exposure, sociodemographics, psychological traits, coping, and social support. The findings highlight the importance of addressing HCWs’ psychological well-being and resilience during and after pandemics. Further research is required to explore PTG in diverse cultural contexts, investigate the dynamic nature of PTG, and evaluate the effectiveness of targeted interventions for HCWs.

### Electronic supplementary material

Below is the link to the electronic supplementary material.

Supplementary Material 1: PRISMA-ScR Checklist

Supplementary Material 2: Database Search Strategy and Description

## Data Availability

All data generated or analyzed during this study are included in this published article [and its supplementary information files].

## Abbreviations

PTG: Posttraumatic growth
VPTG: Vicarious posttraumatic growth
PTGI: Posttraumatic Growth Inventory
PTGI-SF: Short version of the posttraumatic growth inventory
PTGI-X: Expanded posttraumatic growth inventory
COVID‑19: Coronavirus disease 2019
WHO: World Health Organization
HCWs: Healthcare workers
PRISMA‑ScR: Preferred reporting items for systematic reviews and meta‑analyses extension for scoping reviews
PTSD: Posttraumatic stress disorder
PTSS: Posttraumatic stress symptoms
