# Supplementary material for: Posttraumatic growth of medical staff during COVID-19 pandemic: A scoping review
Source: BMC Public Health. 2024 Feb 14;24:460. doi: 10.1186/s12889-023-17591-7 (PMC10865690; doi:10.1186/s12889-023-17591-7)
Supplement: Supplementary file 2 — Supplementary Material 2: Database Search Strategy and Description [file 12889_2023_17591_MOESM2_ESM.docx]

**Database Search Strategy Code and Description**

**Posttraumatic Growth of Medical Staff During COVID-19 Pandemic： A Scoping Review**

*Department of Nursing, The Second Affiliated Hospital of Zhejiang University School of Medicine, China*

*Corresponding author: Lizhu Wang

Address: Department of Nursing, The Second Affiliated Hospital of Zhejiang University School of Medicine. No. 88 Jiefang Road, Shangcheng District, Hangzhou City, Zhejiang Province, China, 310009

E-mail: zrwlz@zju.edu.cn

### PubMed (National Library of Medicine, NCBI)

#1

"COVID-19" OR "SARS-CoV-2" OR "coronavirus disease 2019" OR "con-19" OR "coronavirus disease" OR "2019 n-cov" OR "coronavirus" OR

"COVID-19"[Mesh] OR "SARS-CoV-2"[Mesh]

#2

"Posttraumatic Growth, Psychological"[Mesh] OR "posttraumatic growth" OR "post-traumatic growth" OR "Vicarious Posttraumatic Growth" OR "Secondary Posttraumatic Growth" or "alternative posttraumatic growth"

#3

"Medical Staff"[Mesh] OR "Healthcare workers" OR "HCWs" OR "health workers" OR "health care provider" OR "front line workers" OR "nurse*" OR "doctor*" OR "physician" OR "paramedic" OR "medical workers" OR "medical staff" OR "healthcare professionals" OR "frontline" OR "medical personnel" OR "Health Personnel"[Mesh]

#1 AND #2 AND #3 AND 2020/01/01:2022/12/31[dp]

65 results

### CINAHL (CINAHL Plus with Full Text, Ebsco)

S1

(MH "COVID-19") OR "COVID-19" OR (MH "COVID-19 Pandemic") OR (MH "SARS-CoV-2")

S2

(MH "SARS-CoV-2") OR "SARS-CoV-2" OR (MH "COVID-19")

S3

(MH "Coronavirus Infections") OR "coronavirus disease"

S4

coronavirus or 2019-ncov or sars-cov-2 or cov-19 or 2019 pandemic or pandemic or coronavirus disease or health crisis

S5

(MH "Posttraumatic Growth, Psychological") OR "Posttraumatic Growth"

S6

post traumatic growth or post-traumatic growth or post traumatic growth or ptg

S7

Vicarious Posttraumatic Growth

S8

alternative posttraumatic growth

S9

Secondary Posttraumatic Growth

S10

(MH "Medical Staff") OR "Medical Staff" OR (MH "Medical Staff, Hospital") OR (MH "Health Information Management Personnel") OR (MH "Nursing Staff, Hospital")

S11

(MH "Practical Nurses") OR (MH "Emergency Nurse Practitioners") OR (MH "Gerontologic Nurse Practitioners") OR (MH "OB-GYN Nurse Practitioners") OR "nurse"

S12

(MH "Physicians") OR "doctor"

S13

(MH "Health Personnel") OR "Health Personnel"

S14

( healthcare workers or healthcare professional or healthcare provider or healthcare personnel or doctor or nurse ) OR ( medical workers or medical staff or health care worker or healthcare employee ) OR (front line or front-line or frontline nurses or frontline staff or frontline worker or frontline employee)

S15: S1 OR S2 OR S3 OR S4

S16: S5 OR S6 OR S7 OR S8 OR S9

S17: S10 OR S11 OR S12 OR S13 OR S14

S18: S15 AND S16 AND S17, limit 20200101-20221231

33 results

### PsycINFO (American Psychological Association, Ebsco)

S1

(DE "COVID-19") OR (DE "Coronavirus" OR DE "COVID-19" OR DE "Middle East Respiratory Syndrome" OR DE "Severe Acute Respiratory Syndrome" OR DE "Disease Outbreaks")

S2

DE "COVID-19" OR DE "Coronavirus" OR DE "Disease Outbreaks"

S3

covid-19 or coronavirus or 2019-ncov or sars-cov-2 or cov-19 or 2019 pandemic or pandemic or coronavirus disease or health crisis

S4

DE "Posttraumatic Growth"

S5

post traumatic growth or post-traumatic growth or post traumatic growth or ptg or posttraumatic growth

S6

Secondary Posttraumatic Growth

S7

Vicarious Posttraumatic Growth

S8

alternative posttraumatic growth

S9

DE "Frontline Employees" OR DE "Medical Personnel" OR DE "Dentists" OR DE "Military Medical Personnel" OR DE "Nurses" OR DE "Optometrists" OR DE "Pharmacists" OR DE "Physical Therapists" OR DE "Physicians" OR DE "Psychiatric Hospital Staff" OR DE "Paramedics" OR DE "Health Personnel" OR DE "Allied Health Personnel" OR DE "Caregivers" OR DE "Medical Personnel" OR DE "Mental Health Personnel"

S10

DE "Nurses" OR DE "Psychiatric Nurses" OR DE "Public Health Service Nurses"

S11

DE "Physicians" OR DE "Family Physicians" OR DE "General Practitioners" OR DE "Gynecologists" OR DE "Internists" OR DE "Neurologists" OR DE "Obstetricians" OR DE "Pathologists" OR DE "Pediatricians" OR DE "Psychiatrists" OR DE "Surgeons"

S12

( healthcare workers or healthcare professional or healthcare provider or healthcare personnel or doctor or nurse ) OR ( medical workers or medical staff or health care worker or healthcare employee ) OR (front line or front-line or frontline nurses or frontline staff or frontline worker or frontline employee)

S13: S1 OR S2 OR S3

S14: S4 OR S5 OR S6 OR S7 OR S8

S15: S9 OR S10 OR S11 OR S12

S16: S13 AND S14 AND S15, limit 20200101-20221231

50 results

### Web of Science

#1

"COVID-19" OR "SARS-CoV-2" OR "coronavirus disease 2019" OR "con-19" OR "coronavirus disease" OR "2019 n-cov" OR "coronavirus" OR

"COVID-19"[Mesh] OR "SARS-CoV-2"[Mesh]

#2

"Posttraumatic Growth, Psychological"[Mesh] OR "posttraumatic growth" OR "post-traumatic growth" OR "Vicarious Posttraumatic Growth" OR "Secondary Posttraumatic Growth" or "alternative posttraumatic growth" OR "post traumatic growth" OR PTG

#3

"Medical Staff"[Mesh] OR "Health Personnel"[Mesh] OR healthcare workers OR healthcare professional OR healthcare provider OR healthcare personnel OR doctor OR nurse OR medical workers OR medical staff OR health care worker OR healthcare employee OR front line OR front-line OR frontline nurses OR frontline staff OR frontline worker OR frontline employee

#1 AND #2 AND #3 AND 2020/01/01:2022/12/31[dp]

103 results

### Embase (Elsevier, Embase.com)

PICO

P

'medical staff'/exp OR 'hospital medical staff' OR 'hospitalists' OR 'medical hospital staff' OR 'medical staff' OR 'medical staff, hospital' OR 'staff, medical' OR 'nurse'/exp OR 'anaesthesist nurse assistant' OR 'anesthetist nurse assistant' OR 'community health nurse' OR 'community health nurses' OR 'nurse' OR 'nurse, community health' OR 'nurses' OR 'nurses, community health' OR 'nurses, public health' OR 'nursing assistance' OR 'public health nurse' OR 'public health nurses' OR 'physician'/exp OR 'doctor' OR 'medical doctor' OR 'medical practitioner' OR 'physician' OR 'physician associate' OR 'physicians' OR 'practitioner' OR 'private physician' OR 'health care personnel'/exp OR 'health care personnel' OR 'health care practitioner' OR 'health care professional' OR 'health care provider' OR 'health care worker' OR 'health personnel' OR 'health profession personnel' OR 'health worker' OR 'healthcare personnel' OR 'healthcare practitioner' OR 'healthcare professional' OR 'healthcare provider' OR 'healthcare worker' OR 'home health aides' OR 'personnel, health' OR 'public health officer' OR 'frontline staff'/exp OR 'front line employee' OR 'front line personnel' OR 'front line staff' OR 'front line worker' OR 'frontline employee' OR 'frontline personnel' OR 'frontline staff' OR 'frontline worker' OR 'paramedical personnel'/exp OR 'allied health personnel' OR 'health care assistant' OR 'health care support worker' OR 'health support worker' OR 'healthcare assistant' OR 'healthcare support worker' OR 'ophthalmic assistants' OR 'para medical personnel' OR 'paramedical assistant' OR 'paramedical manpower' OR 'paramedical personnel' OR 'paramedical professional' OR 'paramedical staff' OR 'paramedics' OR 'psychiatric aides' OR 'medical personnel'/exp OR 'district medical officer' OR 'medical corps' OR 'medical officer' OR 'medical personnel' OR 'medical worker' OR 'personnel, medical' OR hcws OR 'frontline'/exp OR 'frontline nurse'/exp OR 'front line nurse' OR 'front line nurses' OR 'frontline nurse' OR 'frontline nurses' OR 'frontline plus'/exp

I

'coronavirus disease 2019'/exp OR '2019 novel coronavirus disease' OR '2019 novel coronavirus epidemic' OR '2019 novel coronavirus infection' OR '2019-ncov disease' OR '2019-ncov infection' OR 'covid' OR 'covid 19' OR 'covid 2019' OR 'covid-10' OR 'covid-19' OR 'covid19' OR 'sars coronavirus 2 infection' OR 'sars-cov-2 disease' OR 'sars-cov-2 infection' OR 'sars-cov2 disease' OR 'sars-cov2 infection' OR 'sarscov2 disease' OR 'sarscov2 infection' OR 'wuhan coronavirus disease' OR 'wuhan coronavirus infection' OR 'coronavirus disease 2' OR 'coronavirus disease 2010' OR 'coronavirus disease 2019' OR 'coronavirus disease-19' OR 'coronavirus infection 2019' OR 'ncov 2019 disease' OR 'ncov 2019 infection' OR 'novel coronavirus 2019 disease' OR 'novel coronavirus 2019 infection' OR 'novel coronavirus disease 2019' OR 'novel coronavirus infection 2019' OR 'paucisymptomatic coronavirus disease 2019' OR 'severe acute respiratory syndrome 2' OR 'severe acute respiratory syndrome cov-2 infection' OR 'severe acute respiratory syndrome coronavirus 2 infection' OR 'severe acute respiratory syndrome coronavirus 2019 infection' OR 'severe acute respiratory syndrome coronavirus 2'/exp OR '2019 ncov' OR '2019 new coronavirus' OR '2019 novel coronavirus' OR '2019 severe acute respiratory syndrome coronavirus 2' OR '2019-ncov' OR 'covid 19 virus' OR 'hcov-19' OR 'human coronavirus 2019' OR 'sars coronavirus 2' OR 'sars coronavirus 2 (ncov-sh01)' OR 'sars-2 (virus)' OR 'sars-2-cov' OR 'sars-cov-2' OR 'sars-cov-2 (ncov-sh01)' OR 'sars-related coronavirus 2' OR 'sars2 (virus)' OR 'sever acute respiratory syndrome coronavirus 2' OR 'severe acute respiratory coronavirus 2' OR 'severe acute respiratory syndorme coronavirus 2' OR 'severe acute respiratory syndrome 2 coronavirus' OR 'severe acute respiratory syndrome coronavirus 2' OR 'severe acute respiratory syndrome coronavirus 2 (ncov-sh01)' OR 'severe acute respiratory syndrome coronoavirus 2' OR 'severe acute respiratory syndrome coronvirus 2' OR 'severe acute respiratory syndrome related coronavirus 2' OR 'severe acute respiratory syndrome virus 2' OR 'severe acute respiratoy syndrome coronavirus 2' OR 'wuhan coronavirus' OR 'wuhan seafood market pneumonia virus' OR 'coronavirus sars-2' OR 'ncov-2019' OR 'novel 2019 coronavirus' OR 'novel coronavirus 2019' OR 'novel coronavirus-19' OR 'severe acute respiratory syndrome 2 virus' OR 'severe acute respiratory syndrome cov-2 virus' OR 'severe acute respiratory syndrome corona virus 2' OR 'severe acute respiratory syndrome coronavirus 2019' OR 'pandemic'/exp OR 'pandemic' OR 'pandemics' OR 'coronavirinae'/exp OR 'coronavirinae' OR 'corona virus' OR 'coronavirus' OR 'coronavirus infection'/exp OR 'coronavirus infection' OR 'coronavirus infections' OR 'corona virus infection' OR 'corona virus infections' OR 'infection by coronavirus' OR 'infection caused by coronavirus' OR 'infection due to coronavirus'

O

'posttraumatic growth (psychology)'/exp OR 'post-traumatic growth (psychology)' OR 'post-traumatic psychological growth' OR 'posttraumatic growth (psychology)' OR 'posttraumatic growth, psychological' OR 'posttraumatic psychological growth' OR 'psychological growth following trauma' OR 'psychological post-traumatic growth' OR 'psychological posttraumatic growth' OR 'posttraumatic growth'/exp OR 'posttraumatic growth inventory'/exp OR 'ptgi posttraumatic growth' OR 'post-traumatic growth inventory' OR 'post-traumatic growth inventory (ptgi)' OR 'posttraumatic growth (ptg) inventory' OR 'posttraumatic growth inventory' OR 'posttraumatic growth inventory (ptgi)' OR 'posttraumatic growth ptg inventory (ptgi)' OR 'post traumatic growth'/exp OR 'posttraumatic growth inventory short form'/exp

P and I and O and [2020-2022]/py

48 results
